# Global prevalence and causes of visual impairment with special reference to the general population of Saudi Arabia

**DOI:** 10.12669/pjms.343.14510

**Published:** 2018

**Authors:** Farhan Khashim Alswailmi

**Affiliations:** 1 Dr. Farhan Khashim Alswailmi, M.D. Faculty of Applied Medical Sciences, University of Hafr Albatin, Hafr Albatin, Kingdom of Saudi Arabia

## Abstract

**Objective:**

This review was undertaken to highlight the worldwide prevalence and causes of visual impairment (VI), on the basis of a wide range of recent and clearly defined data and in comparison with published articles from the Kingdom of Saudi Arabia.

**Methods:**

These data are mainly based on PubMed indexed journal articles. Some representative surveys from each of the six WHO regions across the globe were included in this review with special reference to Saudi Arabian studies.

**Results:**

Published literature show that the prevalence and causes of VI varies markedly in different parts of the world and from region to region within the same country. Cataract, uncorrected refractive errors and glaucoma were shown to be the leading causes of VI worldwide and in Saudi Arabia. Diabetic retinopathy was found to have more contribution in Saudi Arabia due the higher prevalence of diabetes mellitus in this country.

**Conclusion:**

Epidemiological surveys about the prevalence and causes of VI are crucial for the formulation of preventive and curative measures. Data about VI are still scarce with a need to make wider population based surveys, worldwide and in Saudi Arabia for in-depth evaluation of the problem and better strategies to reduce the burden of VI.

## INTRODUCTION

Visual Impairment (VI) is recognized as a global significant health problem which has a serious impact on the personal, economic, and social life of an individual. Population based surveys pertaining to prevalence and causes of VI have already been conducted in more than 65 countries. The absence of surveys and lack of data in the remaining countries have put limitations to the planning, monitoring and evaluation of policies for controlling VI in the world. In Saudi Arabia limited epidemiological surveys pertaining to this problem have been carried out, which indicate that both the prevalence and causes of VI vary in different geographical locations of the country. More population based surveys should be done to evaluate the actual magnitude of this problem in the country.

People can suffer from the problems in their vision at any stage of their life. Most of these problems can be resolved with the use of corrective measures like spectacles or contact lenses. But, some problems in vision may require medical treatment or surgical interventions while as visual acuity in some other conditions cannot be completely recovered even with all of these curative measures. The limitation of the eye(s) or visual system, due to a disease or disorder that may reduce a one’s ability to perform his daily routine activities is referred to as “visual impairment”.^1^ When there is no light perception at all, the condition is referred to as “total blindness”.

The World Health Organization (WHO) has classified levels of VI based either on presenting visual acuity (with present correction) or on the VA achieved with the best possible correction and/or visual field limitation in the better seeing eye. According to the International Classification of Diseases -10 (Update and Revision 2016)^2^ VI is categorized into: Mild or no VI (category 0) for visual acuity (VA) ≥ 6/18, moderate VI (category 1) for VA ≤ 6/18 to ≥ 6/60 and severe VI (category 2) for VA≤ 6/60 to ≥ 3/60 and blindness (category 3, 4 and 5) for VA < 1/60 to no light perception.

## PREVALENCE OF VISUAL IMPAIRMENT

The World Health Organization has estimated that globally about 314 million people are visually impaired and among these the blind people are 45 million.^3^ Despite the fact that that over 80% of global VI is preventable or treatable, millions of people remain at risk of visual loss due to the lack of eye-care services. With almost 90% of blind and visually impaired people living in low- and middle-income countries, including some of the world’s poorest communities, access to eye care is often limited or unavailable. From all visually impaired people around the globe, 19 million alone are children below the age of 15 years.^4^ Most of the world’s blind children live in the poorest regions of Africa and Asia. Visual impairment in children is a severe public health, social, and economic problem worldwide. Results of some representative studies from the six WHO regions around the world are reflected in the Table-I.^5^-^32^

**Table-I:** Prevalence and major cause of VI reported from across the WHO regions.

Region	Country	Age	Sample Size	Prevalence of VI	Main cause of VI	Year of Publication
African Region	Botswana^5^	50 and older	2127	5.38%	CAT	2007
	Cameroon^6^	40 and older	2215	4.4%	CAT	2007
	Equatorial Guinea^7^	1 – 102	3218	14.2%	CAT	2002
	Ghana^8^	16- 39	3437	3.87%	RE	2016
	Kenya^9^	50 and older	2164	13.4%	Not studied	2016
	Nigeria^10^	0 and older	4848	11%	CAT	2011
	Rwanda^11^	50 and older	2206	8.3%	CAT	2007
	Zambia^12^	50 and older	3629	11%	CAT	2012
South-East Asia Region	Bangladesh^13^	0-15	1935	6.9%	CAT	2007
	India^14^	40 and older	6150	15%	CAT	2016
	Indonesia^15^	40 and older	1102	7.72%	RE	2007
	Myanmar^16^	1-99	655	39.5%	CAT	2009
	Nepal^17^	0-10	10769	1.65%	AMB	2015
	Thailand^18^	50 and older	1174	14.5%	CAT	2014
	Timor-Leste^19^	40 and older	1414	29.2%	CAT	2007
Region of the Americas	Brazil^20^	1-91	2485	19.4%	URE	2009
	USA^21^	40 and older	2015 Data	2.82%	RE	2016
European Region	Germany^22^	72+/-22	5100	0.19%	ARMD	2011
	Poland^23^	35 and older	1107	27.5%	ARMD	2015
Eastern Mediterranean Region	Iran^24^	3-93	3095	7.61%	RE	2017
	Pakistan^25^	30 and above	16507	30.4%	CAT	2006
	Saudi Arabia^1^	18 and older	705	23.5%	CAT	2017
	Sudan^26^	05 and above	2499	16.2%	Trachoma	2006
Western Pacific Region	Australia^27^	20 and older	1884	22.2%	RE	2010
	China^28^	60 and older	4190	5.4%	CAT	2017
	Fiji^29^	40 and older	1381	9.8%	CAT	2012
	Japan^30^	40 and older	3762	0.97%	CAT	2010
	Taiwan^31^	65 and older	2316	4.88%	CAT	2012
	Vietnam^32^	12-15	2238	19.4%	RE	2014

*Abbreviations:* AMB; amblyopia, ARMD; age related macular degeneration, RE; refractive error, URE; uncorrected refractive error.

Prevalence of VI was evaluated in various regions of Saudi Arabia and different values for prevalence were reported. The outcome of these studies is summarized in Table-II.^1^ and 33-37 Overall prevalence was estimated to range from 7% to 25.3% in the studied population. Some recent studies in Saudi Arabia were conducted to evaluate the patterns of eye diseases^38^ and patterns of refractive errors.^39^ However VI was not the scope of the research in these studies. Visual impairment is unequally distributed across the different age groups. More than 80% blind people or those who have moderate to severe visual impairment are 50 years of age or older.^40^ Higher incidence of VI due to geriatric diseases has been highlighted by many studies conducted in UK^41^, USA^42^ and Germany.^43^ The prevalence of blindness among children is about 10 times lower than that among adults. However, childhood blindness should remain a high priority because of the expected number of years to be lived in blindness. Globally, the female gender is significantly at higher risk for being visually impaired than males.^33^,^44^,^45^ This higher prevalence is mostly because of their longer life expectancy and in some countries because of their restricted access to the health services due to traditional issues.

**Table-II:** Prevalence and major cause of VI reported from Saudi Arabia.

Area	Age (Years)	Sample size	Prevalence of VI	Major cause of VI	Year of publication	Reference
Arar District Northern Border Region	18 and older	705	23.5%	CAT	2017	1
Aljouf Province Northern Region	18 and older	620	13.9%	RE	2011	33
Riyadh	2 – 18	5217	7%	RE	2005	34
Bisha region	All	2882	11.6%	CAT	1993	35
South Western region	All	1681	25.6%	CAT	1993	36
Al-Baha region	6-18	3590	8.8%	RE	1992	37

Persistence of high prevalence of VI world-wide and Saudi Arabia is questionable as the interventions required are significantly cost effective, and in view of significant improvement in the economic development and quality of life. However, various factors are responsible for refractive errors remaining uncorrected: Lack of awareness and recognition of the problem at personal and family level, as well as at community and public health level; non-availability of and/or inability to afford refractive services for testing; insufficient provision of affordable corrective lenses; and cultural disincentives to compliance.

## CAUSES OF VISUAL IMPAIRMENT

The leading cause of VI and blindness worldwide remains the cataract in both developed and developing countries in spite of the development in its surgical management.^1^,^14^,^18^,^28^,^46^ As people in the world live longer, the number of people with cataract is anticipated to grow.

Congenital cataracts are a very common cause of blindness in the pediatric population. In Saudi Arabia cataract was found to be main cause of VI in Northern region^1^ and South western region.^36^

Uncorrected Refractive Errors (URE) are the main cause of visual impairment and the second leading cause of preventable blindness globally,^47^ while in some recent studies URE is considered as the main leading cause of VI.^8^,^22^,^24^,^32^ In Saudi Arabia URE were reported to be a leading cause of VI in adults of Northern Saudi Arabia^33^ and commonest cause of VI in school children of Qassim Province.^48^ In recent studies from Saudi Arabia higher prevalence of RE have been highlighted in Jazan^38^ and Al Hassa.^49^

Glaucoma is the third leading cause of blindness worldwide. Glaucoma was estimated to be the second major causes of irreversible blindness in the Al Baha province of Saudi Arabia.^50^

Age Related Macular Degeneration (ARMD) ranks as the fourth cause of blindness globally. It has been estimated to be the primary cause of VI in the industrialized countries like Germany^22^ and Poland.^23^ A study from USA,^51^ has indicated that prevalence of VI was significantly higher among people with ARMD than compared to those without ARMD. There is no definite data available on ARMD from Saudi Arabia.

Corneal causes are the major causes of VI after cataract, glaucoma, and age-related macular degeneration. The cause if corneal opacity may be infectious or traumatic. Trachoma is considered leading infectious cause of blindness worldwide especially in under-developed countries with poor water sanitation with higher prevalence among school children. In Saudi Arabia there has been a remarkable decrease in the prevalence of trachoma.^52^ The incidence of trachoma was found to be very low in the western Saudi Arabia^53^ and as well as in the eastern province.^54^ Onchocerciasis is another major infectious cause of blindness in many African countries, Yemen and some countries in Latin America. It is estimated that there are about half a million blind people due to river blindness.^55^ We could find a description of onchocerciasis related sclerosing keratitis in over a dozen of cases seen at the King Faisal Military Hospital in Saudi Arabia.^56^

Diabetic Retinopathy (DR) is the leading cause of blindness among working-age populations in the Western world. DR is more prevalent in people of South Asian, African, Latin American, and indigenous tribal descent compared to the white population.^57^ The alarming rise in the number of diabetic patients in Saudi Arabia has been called an epidemic by many studies. One study has estimated the prevalence of diabetes to be as high as 30% in a Saudi community.^58^ DR was found to be the first major causes of irreversible blindness in Al Baha region^50^ and the third leading cause of VI in Arar City of Saudi Arabia.^25^ DR was estimated to be responsible for 3.3% cases of bilateral blindness in Jazan district of Southern Saudi Arabia.^59^ Higher prevalence of DM and DR has also been indicated in an another study conducted in Taif, Saudi Arabia.^60^

Retinitis Pigmentosa (RP) is the most common cause of inherited blindness. RP was the second commonest cause of low vision in people attending a low vision clinic in Riyadh, Saudi Arabia.^57^ Retinopathy Of Prematurity (ROP) is an important cause in middle-income countries. In pre-term infants, the incidence of ROP was estimated to be 33.7% at two tertiary centers in Jeddah, Saudi Arabia.^62^

The major causes of blindness in children also vary widely from region to region, being mainly determined by socioeconomic development, availability of primary health care and eye care services. In high-income countries, lesions of the optic nerve and higher visual pathways predominate as the cause of blindness, while corneal scarring from measles, vitamin A deficiency, harmful traditional eye remedies, ophthalmia neonatorum, and rubella cataract are the major causes in low-income countries. Other significant causes in all countries are congenital abnormalities, such as cataract, glaucoma, and hereditary retinal dystrophies.

A significant shift, over decades, in the pattern of causes of VI has occurred. The infectious causes have greatly declined over the past two decades. The overall socioeconomic development with improved living conditions in many countries have led to a significant control of communicable causes of blindness, such as trachoma and onchocerciasis.^4^ However these, are still prevalent in the under developed areas. On the other hand, the prevalence of DM is rising with higher position of DR on the priority list of the causes of VI. While some other causes of VI as glaucoma remains on the public health agenda due to difficulties in its early diagnosis and frequent necessity of life long treatment.

## CONCLUSION

There has been an increase in the number of population based surveys pertaining to the prevalence and causes of visual impairment conducted in many countries. However, data about VI are still unidentified in many parts of the world. In Saudi Arabia only six studies have been conducted for VI in different districts, which also indicate that the prevalence and the causes of VI vary from area to area in this country.

Collection of reliable and standardized epidemiological data is a priority for countries, including Saudi Arabia, where such data are limited. Also required is an improved mechanism for systematically collecting standardized information on human resources, infrastructure and available technologies, and countries must be ready to respond to the observed needs.

Both the prevalence and the causes of VI significantly vary from country to country and from region to region. Looking at the previous data one may conclude that final outcome of the studies is greatly affected by a lot of factors including the socioeconomic status, geographical location and efficacy of health care systems of the area where these studies are carried out. In addition the results are expected to be affected by the differences in different types of classifications of VI and also by examination methods used in different studies.

Despite the availability of WHO information on the magnitude and causes of blindness and strategies for their prevention, policy-makers and health providers in some countries are evidently not fully aware of available eye-care interventions, their cost–effectiveness and their potential to prevent or treat the 80% of global blindness that is avoidable. Hence, action is needed to develop modeling approaches in order to determine trends and set targets, so that the planning of efforts to prevent avoidable blindness and visual impairment can be more focused and evidence-based.

Unless additional eye-care services are provided, the number of people suffering from vision loss due to chronic age-related eye diseases will rise as a result of increased life expectancy and population growth.

Screening of children for refractive errors should be conducted at community level and integrated into school health programs, accompanied by education and awareness campaigns to ensure that the corrections are used and cultural barriers to compliance are addressed and removed.

### Abbreviations

**ARMD:** Age Related Macular Degeneration.

**DR:** Diabetic Retinopathy.

**RE:** Refractive Error.

**ROP:** Retinopathy of Prematurity.

**RP:** Retinitis Pigmentosa.

**VI:** Visual Impairment.
